# 4-(1*H*-Benzimidazol-2-yl)benzonitrile

**DOI:** 10.1107/S1600536808012932

**Published:** 2008-05-07

**Authors:** Wei Dai, Wen-Xiang Wang, Yu-Yuan Zhao, Hong Zhao

**Affiliations:** a Ordered Matter Science Research Center, College of Chemistry and Chemical Engineering, Southeast University, Nanjing 210096, People’s Republic of China

## Abstract

The mol­ecule of the title compound, C_14_H_9_N_3_, is essentially planar, the dihedral angle formed by the benzimidazole ring system with the benzene ring being 3.87 (3)°. In the crystal packing, mol­ecules are linked into zigzag chains running parallel to the *b* axis by inter­molecular N—H⋯N hydrogen-bond inter­actions.

## Related literature

For related literature, see: Gallagher *et al.* (2001 ▶); Howarth & Hanlon (2001 ▶); Kazak *et al.* (2006 ▶); Li *et al.* (1998 ▶); Íkizler & Sancak (1992 ▶).
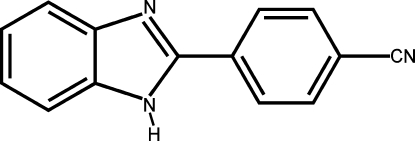


## Experimental

### 

#### Crystal data

C_14_H_9_N_3_
*M* *_r_* = 219.24
Monoclinic, 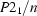

*a* = 7.2172 (10) Å
*b* = 11.818 (2) Å
*c* = 12.719 (2) Å
β = 92.057 (7)°
*V* = 1084.1 (3) Å^3^
*Z* = 4
Mo *K*α radiation
μ = 0.08 mm^−1^
*T* = 293 (2) K
0.35 × 0.15 × 0.10 mm

#### Data collection

Rigaku Mercury2 diffractometer
Absorption correction: multi-scan (*CrystalClear*; Rigaku, 2005 ▶) *T*_min_ = 0.910, *T*_max_ = 1.000 (expected range = 0.903–0.992)
11203 measured reflections
2581 independent reflections
2073 reflections with *I* > 2σ(*I*)
*R*_int_ = 0.037

#### Refinement

*R*[*F* ^2^ > 2σ(*F* ^2^)] = 0.045
*wR*(*F* ^2^) = 0.118
*S* = 1.08
2581 reflections
159 parameters
H atoms treated by a mixture of independent and constrained refinement
Δρ_max_ = 0.16 e Å^−3^
Δρ_min_ = −0.17 e Å^−3^

### 

Data collection: *CrystalClear* (Rigaku, 2005 ▶); cell refinement: *CrystalClear*; data reduction: *CrystalClear*; program(s) used to solve structure: *SHELXS97* (Sheldrick, 2008 ▶); program(s) used to refine structure: *SHELXL97* (Sheldrick, 2008 ▶); molecular graphics: *SHELXTL* (Sheldrick, 2008 ▶); software used to prepare material for publication: *SHELXL97*.

## Supplementary Material

Crystal structure: contains datablocks I, global. DOI: 10.1107/S1600536808012932/rz2211sup1.cif

Structure factors: contains datablocks I. DOI: 10.1107/S1600536808012932/rz2211Isup2.hkl

Additional supplementary materials:  crystallographic information; 3D view; checkCIF report

## Figures and Tables

**Table 1:** Hydrogen-bond geometry (Å, °)

*D*—H⋯*A*	*D*—H	H⋯*A*	*D*⋯*A*	*D*—H⋯*A*
C13—H13*A*⋯N1	0.93	2.54	2.861 (2)	101
N2—H2*A*⋯N3^i^	0.910 (17)	2.14 (2)	3.033 (2)	169.1 (15)

Symmetry code: (i) 
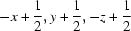
.

**Fig. 1.**
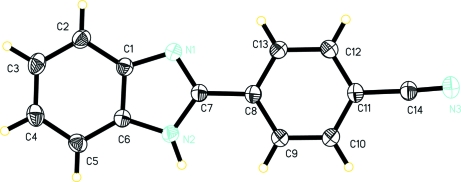
The molecular structure of the title compound with the atom-numbering scheme. Displacement ellipsoids are drawn at the 30% probability level.

## supplementary crystallographic information

### Comment

Benzimidazole systems continue to attract much attention in chemical synthesis, structural science and applied biological research (Li *et al.*, 1998; Gallagher *et al.*, 2001; Howarth & Hanlon, 2001; Kazak *et al.*, 2006). Nitriles are parent compounds for the preparation of various functional organic materials having triazole, imidazole or thidiazole moieties (Íkizler & Sancak, 1992) and their derivatives have found many industrial applications. We report here the crystal structure of the title compound, 4-(1*H*-benzo[*d*]imidazol-2-yl) benzonitrile.

The structural analysis shows that in the title compound (Fig. 1) the benzimidazole ring system and the phenyl ring are nearly coplanar, the dihedral angle they form being 3.87 (3)°. In the imidazole ring, the C7δb N1 bond length of 1.3191 (16) Å conforms to the value for a double bond. The molecular conformation is stabilized by an intramolecular C—H..N hydrogen bond (Table 1). In the crystal structure, molecules are linked into zig-zag chains running parallel to the b axis by intermolecular N—H···N hydrogen bonding interactions involving the protonated N atom of the imidazole ring as H-donor and the N atom of the nitrile group as acceptor.

### Experimental

4-Formylbenzonitrile (2 mmol), malononitrile (1 mmol) and benzene-1,2-diamine (1 mmol) were heated at 100°C with stirring for 5 min. The mixture was washed with dichloromethane(5 mL) and dried. A white solid was obtained after recrystallization from ethanol. 4-(1*H*-Benzo[*d*]imidazol-2-yl)benzonitrile (0.3 mmol) was placed in a thick-walled Pyrex tube. EtOH (0.3 mL) and H_2_O (0.3 mL) were then added, the tube was frozen with liquid N_2_, evacuated and flame-sealed. The tube was heated at 100°C for 2 days to give colourless crystals of the title compound.

### Refinement

The H atom bound to the imidazole N atom was located in a difference Fourier synthesis and refined freely. All other H atoms were placed in calculated positions and refined using a riding model approximation, with C—H = 0.93 Å and *U*_iso_(H) = 1.2 *U*_eq_(C).

### Crystal data

**Table:**

C_14_H_9_N_3_	*F*_000_ = 456
*M**_r_* = 219.24	*D*_x_ = 1.343 Mg m^−^^3^
Monoclinic, *P*2_1_/*n*	Mo *K*α radiation λ = 0.71073 Å
Hall symbol: -P 2yn	Cell parameters from 2461 reflections
*a* = 7.2172 (10) Å	θ = 3.2–27.5º
*b* = 11.818 (2) Å	µ = 0.08 mm^−^^1^
*c* = 12.719 (2) Å	*T* = 293 (2) K
β = 92.057 (7)º	Prism, colourless
*V* = 1084.1 (3) Å^3^	0.35 × 0.15 × 0.10 mm
*Z* = 4	

### Data collection

**Table:**

Rigaku Mercury2 (2x2 bin mode) diffractometer	2581 independent reflections
Radiation source: fine-focus sealed tube	2073 reflections with *I* > 2σ(*I*)
Monochromator: graphite	*R*_int_ = 0.037
Detector resolution: 13.6612 pixels mm^-1^	θ_max_ = 27.9º
*T* = 293(2) K	θ_min_ = 2.4º
CCD_Profile_fitting scans	*h* = −9→9
Absorption correction: multi-scan(CrystalClear; Rigaku, 2005)	*k* = −15→15
*T*_min_ = 0.911, *T*_max_ = 1.000	*l* = −16→16
11203 measured reflections	

### Refinement

**Table:**

Refinement on *F*^2^	Hydrogen site location: inferred from neighbouring sites
Least-squares matrix: full	H atoms treated by a mixture of independent and constrained refinement
*R*[*F*^2^ > 2σ(*F*^2^)] = 0.046	*w* = 1/[σ^2^(*F*_o_^2^) + (0.056*P*)^2^ + 0.1336*P*] where *P* = (*F*_o_^2^ + 2*F*_c_^2^)/3
*wR*(*F*^2^) = 0.118	(Δ/σ)_max_ < 0.001
*S* = 1.08	Δρ_max_ = 0.16 e Å^−^^3^
2581 reflections	Δρ_min_ = −0.17 e Å^−^^3^
159 parameters	Extinction correction: SHELXL97 (Sheldrick, 2008), Fc^*^=kFc[1+0.001xFc^2^λ^3^/sin(2θ)]^-1/4^
Primary atom site location: structure-invariant direct methods	Extinction coefficient: 0.115 (7)
Secondary atom site location: difference Fourier map	

### Special details

**Table:**

Geometry. All e.s.d.'s (except the e.s.d. in the dihedral angle between two l.s. planes) are estimated using the full covariance matrix. The cell e.s.d.'s are taken into account individually in the estimation of e.s.d.'s in distances, angles and torsion angles; correlations between e.s.d.'s in cell parameters are only used when they are defined by crystal symmetry. An approximate (isotropic) treatment of cell e.s.d.'s is used for estimating e.s.d.'s involving l.s. planes.
Refinement. Refinement of *F*^2^ against ALL reflections. The weighted *R*-factor *wR* and goodness of fit *S* are based on *F*^2^, conventional *R*-factors *R* are based on *F*, with *F* set to zero for negative *F*^2^. The threshold expression of *F*^2^ > σ(*F*^2^) is used only for calculating *R*-factors(gt) *etc*. and is not relevant to the choice of reflections for refinement. *R*-factors based on *F*^2^ are statistically about twice as large as those based on *F*, and *R*- factors based on ALL data will be even larger.

### Fractional atomic coordinates and isotropic or equivalent isotropic displacement parameters (Å^2^)

**Table:**

	*x*	*y*	*z*	*U*_iso_*/*U*_eq_	
N2	0.23276 (15)	0.98417 (9)	−0.07818 (9)	0.0381 (3)	
C8	0.14908 (17)	0.84634 (11)	0.06171 (10)	0.0367 (3)	
N1	0.08189 (15)	0.82641 (9)	−0.12784 (8)	0.0404 (3)	
C7	0.15245 (17)	0.88501 (10)	−0.04772 (10)	0.0360 (3)	
N3	0.11261 (18)	0.68424 (11)	0.45407 (10)	0.0543 (3)	
C11	0.13598 (18)	0.76582 (11)	0.26664 (10)	0.0389 (3)	
C6	0.21340 (17)	0.98948 (10)	−0.18604 (10)	0.0363 (3)	
C9	0.23427 (19)	0.90578 (12)	0.14485 (10)	0.0439 (3)	
H9A	0.2963	0.9730	0.1315	0.053*	
C1	0.11833 (17)	0.89042 (11)	−0.21590 (10)	0.0377 (3)	
C5	0.2689 (2)	1.06900 (11)	−0.25904 (11)	0.0443 (3)	
H5A	0.3300	1.1351	−0.2384	0.053*	
C14	0.12508 (18)	0.72221 (12)	0.37201 (11)	0.0425 (3)	
C13	0.05688 (19)	0.74595 (12)	0.08336 (11)	0.0432 (3)	
H13A	−0.0015	0.7058	0.0287	0.052*	
C12	0.05122 (19)	0.70550 (12)	0.18459 (11)	0.0436 (3)	
H12A	−0.0093	0.6378	0.1980	0.052*	
C10	0.2277 (2)	0.86617 (12)	0.24669 (11)	0.0459 (3)	
H10A	0.2846	0.9066	0.3017	0.055*	
C4	0.2292 (2)	1.04531 (13)	−0.36315 (11)	0.0496 (4)	
H4A	0.2652	1.0964	−0.4142	0.060*	
C3	0.1362 (2)	0.94670 (13)	−0.39450 (11)	0.0492 (4)	
H3A	0.1134	0.9332	−0.4658	0.059*	
C2	0.07759 (19)	0.86882 (12)	−0.32224 (10)	0.0451 (3)	
H2B	0.0131	0.8041	−0.3435	0.054*	
H2A	0.281 (2)	1.0377 (15)	−0.0336 (13)	0.058 (5)*	

### Atomic displacement parameters (Å^2^)

**Table:**

	*U*^11^	*U*^22^	*U*^33^	*U*^12^	*U*^13^	*U*^23^
N2	0.0439 (6)	0.0349 (6)	0.0354 (6)	0.0001 (4)	0.0010 (5)	−0.0012 (4)
C8	0.0358 (6)	0.0391 (7)	0.0352 (7)	0.0032 (5)	0.0033 (5)	0.0003 (5)
N1	0.0455 (6)	0.0407 (6)	0.0349 (6)	−0.0031 (5)	−0.0001 (4)	0.0003 (4)
C7	0.0377 (6)	0.0355 (6)	0.0350 (7)	0.0035 (5)	0.0025 (5)	0.0001 (5)
N3	0.0641 (8)	0.0563 (8)	0.0425 (7)	−0.0005 (6)	0.0002 (6)	0.0094 (6)
C11	0.0391 (7)	0.0420 (7)	0.0357 (7)	0.0050 (5)	0.0029 (5)	0.0042 (5)
C6	0.0382 (6)	0.0351 (7)	0.0357 (7)	0.0050 (5)	0.0020 (5)	0.0003 (5)
C9	0.0535 (8)	0.0389 (7)	0.0393 (8)	−0.0058 (6)	−0.0006 (6)	0.0025 (5)
C1	0.0394 (6)	0.0375 (7)	0.0360 (7)	0.0042 (5)	−0.0002 (5)	0.0023 (5)
C5	0.0502 (8)	0.0360 (7)	0.0468 (8)	0.0010 (6)	0.0028 (6)	0.0046 (6)
C14	0.0468 (8)	0.0425 (7)	0.0381 (7)	0.0035 (5)	0.0011 (6)	0.0026 (5)
C13	0.0463 (7)	0.0464 (8)	0.0369 (7)	−0.0059 (6)	0.0000 (5)	−0.0026 (6)
C12	0.0461 (7)	0.0434 (7)	0.0414 (7)	−0.0061 (6)	0.0034 (6)	0.0025 (6)
C10	0.0549 (8)	0.0448 (8)	0.0376 (7)	−0.0049 (6)	−0.0038 (6)	−0.0009 (6)
C4	0.0590 (9)	0.0472 (8)	0.0430 (8)	0.0079 (6)	0.0050 (6)	0.0122 (6)
C3	0.0611 (9)	0.0508 (8)	0.0354 (7)	0.0113 (7)	−0.0042 (6)	0.0050 (6)
C2	0.0519 (8)	0.0439 (7)	0.0390 (7)	0.0023 (6)	−0.0058 (6)	−0.0016 (6)

### Geometric parameters (Å, °)

**Table:**

N2—C7	1.3696 (16)	C9—C10	1.3798 (19)
N2—C6	1.3754 (17)	C9—H9A	0.9300
N2—H2A	0.910 (17)	C1—C2	1.3972 (18)
C8—C13	1.3926 (19)	C5—C4	1.374 (2)
C8—C9	1.3940 (19)	C5—H5A	0.9300
C8—C7	1.4661 (17)	C13—C12	1.3756 (18)
N1—C7	1.3191 (16)	C13—H13A	0.9300
N1—C1	1.3846 (16)	C12—H12A	0.9300
N3—C14	1.1427 (17)	C10—H10A	0.9300
C11—C10	1.3861 (19)	C4—C3	1.396 (2)
C11—C12	1.3877 (19)	C4—H4A	0.9300
C11—C14	1.4407 (18)	C3—C2	1.378 (2)
C6—C5	1.3901 (18)	C3—H3A	0.9300
C6—C1	1.4025 (18)	C2—H2B	0.9300
			
C7—N2—C6	107.00 (11)	C4—C5—C6	116.80 (13)
C7—N2—H2A	125.0 (10)	C4—C5—H5A	121.6
C6—N2—H2A	127.8 (10)	C6—C5—H5A	121.6
C13—C8—C9	118.70 (12)	N3—C14—C11	177.40 (16)
C13—C8—C7	118.53 (12)	C12—C13—C8	120.77 (13)
C9—C8—C7	122.77 (12)	C12—C13—H13A	119.6
C7—N1—C1	105.01 (11)	C8—C13—H13A	119.6
N1—C7—N2	112.72 (11)	C13—C12—C11	119.89 (13)
N1—C7—C8	123.42 (12)	C13—C12—H12A	120.1
N2—C7—C8	123.84 (11)	C11—C12—H12A	120.1
C10—C11—C12	120.16 (12)	C11—C10—C9	119.67 (13)
C10—C11—C14	121.25 (12)	C11—C10—H10A	120.2
C12—C11—C14	118.59 (12)	C9—C10—H10A	120.2
N2—C6—C5	132.50 (12)	C5—C4—C3	121.81 (13)
N2—C6—C1	105.27 (11)	C5—C4—H4A	119.1
C5—C6—C1	122.23 (12)	C3—C4—H4A	119.1
C10—C9—C8	120.80 (13)	C2—C3—C4	121.54 (14)
C10—C9—H9A	119.6	C2—C3—H3A	119.2
C8—C9—H9A	119.6	C4—C3—H3A	119.2
N1—C1—C2	130.07 (12)	C3—C2—C1	117.69 (13)
N1—C1—C6	110.01 (11)	C3—C2—H2B	121.2
C2—C1—C6	119.91 (12)	C1—C2—H2B	121.2

### Hydrogen-bond geometry (Å, °)

**Table:**

*D*—H···*A*	*D*—H	H···*A*	*D*···*A*	*D*—H···*A*
C13—H13A···N1	0.93	2.54	2.861 (2)	101
N2—H2A···N3^i^	0.910 (17)	2.14 (2)	3.033 (2)	169.1 (15)

Symmetry codes: (i) −*x*+1/2, *y*+1/2, −*z*+1/2.
